# Mammographic screening for young women with a family history of breast cancer: knowledge and views of those at risk

**DOI:** 10.1038/sj.bjc.6604672

**Published:** 2008-09-30

**Authors:** S Tyndel, A Clements, C Bankhead, B J Henderson, K Brain, E Watson, J Austoker

**Affiliations:** 1 Department of Primary Health Care, University of Oxford, Old Road, Headington, Oxford OX3 7LF, UK; 2 CRUK Primary Care Education Research Group, Cancer Epidemiology Unit, University of Oxford, Richard Doll Building, Roosevelt Drive, Oxford OX3 7DG, UK; 3 School of Psychology, Brigantia Building, Bangor University, Bangor, Gwynedd, LL57 2AS, UK; 4 Clinical Epidemiology Interdisciplinary Research Group, Institute of Medical Genetics, School of Medicine, Cardiff University, Heath Park, Cardiff CF14 4XN, UK; 5 School of Health and Social Care, Oxford Brookes University, Jack Straws Lane, Marston, Oxford OX3 0FL, UK

**Keywords:** mammography, family history of breast cancer, under 50, informed, knowledge, views

## Abstract

Although the effectiveness of mammography for women under the age of 50 years with a family history of breast cancer (FHBC) has not yet been proven, annual screening is being offered to these women to manage breast cancer risk. This study investigates women's awareness and interpretation of their familial risk and knowledge and views about mammographic screening. A total of 2231 women from 21 familial/breast/genetics centres who were assessed as moderate risk (17–30% lifetime risk) or high risk (>30% lifetime risk) completed a questionnaire before their mammographic screening appointment. Most women (70%) believed they were likely, very likely or definitely going to develop breast cancer in their lifetime. Almost all women (97%) understood that the purpose of mammographic screening was to allow the early detection of breast cancer. However, 20% believed that a normal mammogram result meant there was definitely no breast cancer present, and only 4% understood that screening has not been proven to save lives in women under the age of 50 years. Women held positive views on mammography but did not appear to be well informed about the potential disadvantages. These findings suggest that further attention should be paid to improving information provision to women with an FHBC being offered routine screening.

In the United Kingdom, as part of the national screening programme, routine mammographic screening is only offered to women aged over 50 years. However, women aged under 50 years who are assessed as having a moderate (17–30% lifetime risk) or high (>30% lifetime risk) risk of developing breast cancer are offered annual mammograms. Some women will be eligible for genetic testing, but the majority of women with a family history of breast cancer (FHBC) will have their risk managed through annual mammographic screening as recommended by management guidelines (National Institute for Clinical Excellence, 2004).

It has been increasingly recognised as important that individuals make an informed choice regarding whether or not to participate in screening (National Screening Committee, 2000). For women with an FHBC, being informed means knowing not only what having an increased risk means in terms of how likely they are to develop breast cancer but also knowing about the advantages and disadvantages of screening before 50 years of age. However, many women with an FHBC may not have been fully informed. For example, it has been reported that recall of risk level may be inaccurate after counselling about the risk of developing breast cancer (Hopwood *et al*, 2003) and that inaccuracies in risk perception may persist (Leventhal *et al*, 1999; Hopwood *et al*, 2003; d'Agincourt-Canning, 2005). Furthermore, studies of UK women aged over 50 years in routine screening (Webster and Austoker, 2006) other general populations (Woloshin *et al*, 2000; Chamot and Perneger, 2001; Silverman *et al*, 2001; Domenighetti *et al*, 2003) and in women aged under 50 years (Black *et al*, 1995; Nekhlyudov *et al*, 2003) have reported that women's knowledge about mammography is variable and sometimes incorrect.

To our knowledge, this study is the first large, multicentre UK study to elicit the views and knowledge of women with an FHBC regarding mammographic screening. The following key questions are addressed: (1) What do women know about their level of breast cancer risk? (2) What do women know about the purpose, possible consequences and the effectiveness of mammography? (3) What are women's views about having a mammogram?

## Materials and methods

### Participants

A total of 3740 women were invited to participate in the study if they were aged 35–49 years and had been accepted on an annual screening programme after they had been assessed at specialist family/breast/genetics clinics as being at moderate or high risk of familial breast cancer. Women were excluded if they had a previous diagnosis of breast cancer, an ovarian only family history or were deemed to be low risk.

Familial risk assessment and acceptance on a family history programme followed a similar pattern in the clinics participating in the study. The women were referred to specialist clinics by their general practitioner (GP) or by hospital consultants (e.g., breast surgeons). All women referred to the clinics were asked to complete a family history questionnaire, which was used to assess risk levels. Assessment into a moderate or high risk category was in accordance with the NICE guidelines published in 2004 (National Institute for Clinical Excellence, 2004). Both moderate and high-risk women were offered regular mammograms at 1 year to 18-month intervals and were counselled by a health professional from the specialist clinics. In addition, all high-risk women were offered referral to a genetics specialist for counselling. If eligible or appropriate, high-risk women could also be offered genetic testing or risk-reducing prophylactic surgery.

### Procedures

Twenty-one centres running clinics for women with an FHBC throughout the United Kingdom sent questionnaires with a reply paid envelope to women 1 month (range 2 weeks to 4 months) before their scheduled mammogram appointment. Where time permitted, centres sent a reminder to women who did not return their questionnaire within 2 weeks.

Women who missed one appointment and failed to contact the clinic within 2 months, or those that missed two or more appointments were classified as non-attenders. These women were sent a brief non-attendance questionnaire to elicit their reasons for not attending.

The London Multicentre Research Ethics Committee approved the study.

### Measures

*The questionnaire included the following items* Socio-demographic measures Age, marital status, ethnicity, educational level, number of biological children and menopausal status.

*Screening history* Women were asked whether they had had previous mammograms or previous recall for further tests.

*Family history* Screening centres categorised women as either moderate or high risk. Women provided information on whether a first-degree family member had died of breast cancer or was currently being treated for breast cancer.

*Concerns about breast cancer* Cancer Worry Scale Revised (Lerman *et al*, 1991a, 1991b; Watson *et al*, 1998). This six item questionnaire assesses the degree of worry about developing cancer and the impact of worry on daily functioning in the previous month (Hopwood *et al*, 2001; Brain *et al*, 2002; Fry *et al*, 2003; Henderson *et al*, 2008).

The Psychological Consequences Questionnaire (Cockburn *et al*, 1992) is a 12-item questionnaire, which measures negative consequences of breast screening in terms of emotional, physical and social functioning in the previous month (Ong *et al*, 1997; Tyndel *et al*, 2007).

*Awareness of being at risk of breast cancer* Women were asked when they first realised they were at risk of breast cancer. They were also asked ‘Have you been told what your level of risk of developing breast cancer is?’ (no/don't know/yes) and ‘if yes, what were you told your level of risk is?’ (low/moderate/high or please describe in your own terms).

*Risk perception* Perception of likelihood of breast cancer was measured by asking women ‘how likely do you think it is that you will develop breast cancer in your lifetime?’ The question was scored from 1 (definitely) to 5 (no chance). Responses were dichotomised by combining ‘definitely, highly likely and likely’ and ‘unlikely, no chance’ for analysis.

Perception of comparative risk of breast cancer was measured by asking women ‘Compared to most other women your age, do you think your own chances of getting breast cancer at some time in you life are?’ The question was scored from 1 (much more) to 5 (much less). Both these questions were developed from previous research (Hopwood *et al*, 2003). Responses were dichotomised by combining ‘much more, a little more’ and ‘about the same, a little less, much less’ for analysis.

*Knowledge* Women were asked several questions to gauge their knowledge about the purpose, possible consequences and effectiveness of mammography. There were six multiple choice response options for each question, apart from the last item that consisted of three. Women were also given the option to respond ‘don't know’ to any of the questions. Responses to the questions were scored as either correct or incorrect. Correct responses are given in brackets. The questions were as follows:

1. What do you think the main purpose of breast screening (mammography) is? (*to allow the early detection of breast cancer*).
2. Of women who go along for a routine screening mammography about how many do you think will be given a normal result (*most of them*).
3. If, after a mammogram, a woman is given a normal result what do you think this means? (*highly unlikely or unlikely breast cancer is present*).
4. After a mammogram, women may be called back because the appearance of the x-ray suggests further tests are needed. How often do you think this happens? (*sometimes*).
5. Of the women who are called back for further tests after their mammogram, about how many will turn out to have breast cancer (*some or a few*).
6. Mammography has been proven to save lives from breast cancer in women under the age of 50 years with a family history like yours (*false*)

*Views on mammographic screening* Attitudes towards mammographic screening were measured using an eight-item scale. The questionnaire aimed to measure both positive and negative attitudes to mammography. Women were asked how much they agreed with four positive statements such as ‘Having breast screening (mammography) would make me feel that I am doing something positive about my risk of breast cancer’ and four negative statements such as ‘Having breast screening (mammography) would be a frightening experience for me’. Items are scored from 1 to 3 (strongly agree/agree), 4 (neither agree nor disagree) and 5–7 (disagree/strongly disagree). The eight items were factors analysed using principal components analysis, which revealed two components: a positive (Cronbach's *α*=0.72) and a negative (Cronbach's *α*=0.54). Responses to the positive component were dichotomised into agree (4–12) and neutral/disagree (13–28) for further analysis. The negative component was not analysed further because of the low Cronbach *α*-score.

*Reasons for non-attendance* Women were presented with 21 statements of reasons for not attending the clinics plus an option to add their own views and were also asked to identify up to three main reasons for not attending.

### Statistical analysis

A descriptive analysis was conducted to explore women's knowledge and views. Percentages and 95% confidence intervals are presented. Further exploratory analyses to investigate differences between groups were conducted using the *χ*^2^ test and *t*-test as appropriate. The Yates continuity correction was used when comparisons were made between dichotomised variables. Age and cancer worry scores (CWS-R, PCQ) were treated as continuous variables. Owing to the large number of comparisons being made, only results that reached a level of significance of <0.01 were considered statistically significant.

## Results

### Study participants

After ineligible women were excluded, that is, those who completed the questionnaire after their mammogram or >4 months before their mammogram (244), or did not fulfil the inclusion criteria (222), 2321 women completed the pre-screening questionnaire giving a response rate of 62% (2321 out of 3740). In this study, 52 out of 2321 (2%) who completed the pre-screening questionnaire did not attend their mammogram appointment. The sociodemographic, screening history, family history and psychological variables of the whole sample are listed in Table 1.

### Beliefs about risk and likelihood of developing breast cancer

*Awareness of breast cancer risk* Of 439 women participating in the programme for the first time, 38% (95% CI: 33.9–43.2) reported being aware that they were at increased risk >5 years ago. Seventeen per cent (95% CI: 13.9–21.2%) of women reported realising they were at risk <6 months ago.

*Knowledge of level of breast cancer risk* Out of the total cohort, 73% (95% CI: 71–74.7) reported they had been told their risk level and 63% (95% CI: 60.5–64.5) reported what they had been told. Of the 1435 women who reported their risk, compared with clinic-risk level, 69% (95% CI: 66.0–70.9) reported their risk correctly. Of the 782 moderate-risk women, 17% (95% CI: 14.8–20.2) underestimated their risk, 16% (95% CI: 13.4–18.6) overestimated and 67% (95% CI: 63.3–70) reported their risk correctly. Of the 653 high-risk women, 71% (95% CI: 66.9–74.1) correctly reported their risk and 29% (95% CI: 25.9–33.1) underestimated their risk.

Of the 1731 women who had been attending a screening programme for more than 1 year, 27% (95% CI: 24.4–28.6) reported they had not been told their risk or that they did not know if they had been told their risk level. These women were significantly less likely to have had higher (college or university) education (26 *vs* 34%; *P*<0.000) and significantly less likely to be high risk (28 *vs* 48%; *P*<0.000) than the women who had been told their risk.

*Perceived risk of developing breast cancer* When asked how likely they think it was that they would develop breast cancer in their lifetime, 20% (95% CI: 18.3–21.5) of the women reported not knowing. This was significantly related to whether women reported being told their risk (35 *vs* 25%; *P*<0.001) and these women also tended to have lower levels of education (77 *vs* 67%; *P*<0.001) and to be moderate risk (68 *vs* 56%; *P*<0.001).

Of the 1826 women in the total cohort who believed they did know their likelihood of developing breast cancer, 87% (95% CI: 85.8–88.9) believed they may develop the disease. Comparisons between high-risk and moderate-risk women showed that those at high risk were significantly more sure of the likelihood of developing breast cancer (93 *vs* 83%; *P*<0.001). Two per cent (95% CI: 1.8–3.1) of the women believed they would definitely develop cancer. There were no significant differences in whether these women had been assessed as moderate or high risk or whether the women had been told their actual risk level. But as these numbers are very small, these results should be interpreted with caution.

Women were also asked to rate their own chances of developing breast cancer compared with most other women their age. Of the 2210 women who answered the question, 87% (95% CI: 85.3–88.1) believed that their chances of getting cancer were ‘a little more’ to ‘much more’ than most other women of their age and high-risk women were significantly more sure that their risk was higher than other women (93 *vs* 83%; *P*<0.001).

### Knowledge of purpose, possible consequences and effectiveness of mammography

Almost all women (97%; 95% CI: 96.6–97.9) reported that the main purpose of mammography is the early detection of breast cancer. The majority of women (75%; 95% CI: 72.7–76.3) knew that most women would be given a normal result and 77% (95% CI: 75.6–77.1) knew that following a mammogram, women were ‘sometimes’ recalled for further tests. Similar numbers overestimated recall (9%; 95% CI: 8.1–10.5) as underestimated it (7%; 95% CI: 5.7–7.8). Eighty-six per cent (95% CI: 84.5–87.4) of women knew that not all women recalled would be diagnosed with cancer. A small number (4%; 95% CI: 3.4–5.1) thought incorrectly that ‘most’ women recalled would be diagnosed with cancer. Only 4% (95% CI: 3.0–4.6) of women knew that mammography had not been proven to save lives from breast cancer in women under the age of 50 years with a family history and 20% (95% CI: 18.7–22) of women thought that a normal result after a mammogram meant that there was definitely no breast cancer present.

### Views on mammography

Ninety per cent (95% CI: 88.7–91.2) of women felt that having a mammogram would reassure them that everything was OK and 96% (95% CI: 95–96.7) felt that having a mammogram would make them feel they were doing something positive. Eighty-one per cent (95% CI: 79.3–82.5) of women felt that having a mammogram would make them feel less anxious about breast cancer and 78% (95% CI: 76.6–80.1) felt that it would reduce their chances of dying of breast cancer. However, 35% (95% CI: 32.5–36.5) felt that mammograms would be painful, 14% (95% CI: 12.7–15.6) felt that having a mammogram would be frightening, 16% (95% CI: 14.6–17.7) felt that having a mammogram would make them worry about the effects of radiation and 8% (95% CI: 6.5–8.7) felt that having a mammogram would make them worry unnecessarily.

Most of the women were positive about mammography. However, 12% (95% CI: 10.9–13.7) of women scored highly on the positive scale, indicating a greater level of disagreement. There were no significant differences between baseline characteristics of these women (cutoff *P*<0.01). But, these women were more distressed (CWS: 12.2 *vs* 11.1: *P*=0.000 and PCQ: 8.4 *vs* 5.6; *P*=0.000). They were also more likely be incorrect about how many women would have a normal result (33 *vs* 24%; *P*=0.001), more likely to be correct about what a normal result means (86 *vs* 78%; *P*=0.006), be more pessimistic about how many recalled women would be diagnosed with cancer (20 *vs* 13%; *P*=0.002) and believe that mammography has not been proven to save lives (8 *vs* 3%; *P*=0.000).

### Reasons for non-attendance at the screening appointment

Fifty-four per cent (95% CI: 39.5–67.8) completed a questionnaire on reasons for non-attendance. Table 2 gives the frequency of response to different reasons for non-attendance. It also shows what the main reasons were for not attending for mammography. The main reasons given were practical with the reason given by the most number of women being ‘having problems getting to the breast care unit’. Only three women reported that being anxious about the possible outcome was a main barrier to attendance.

### Summary of results

Most women had been aware of their increased risk for several years before attending the screening programme. Not all women reported that they had been told their risk level and of those who did, the majority reported the level correctly, but a substantial minority reported it incorrectly. Regardless of the level of risk, most women believed that they may develop breast cancer in their lifetime. Women in this cohort were very motivated to attend screening and the majority had positive views about mammographic screening. The women who had less positive views about mammography tended to report higher cancer-specific distress. The majority of women responded correctly to the questions about the purpose and possible consequences of mammography. However, some were incorrect about the sensitivity of mammography, and nearly all were incorrect in believing that mammography had been proven to decrease mortality in women under the age of 50 years. Comparisons of the relationship between views and knowledge showed that the women who were less positive about mammography were more likely to underestimate a good outcome.

## Discussion

Even before attending a familial breast clinic, many of the women in this study had been aware that they may be at risk of developing breast cancer for several years, and it has been suggested that obtaining information about personal risk is a primary motivation for attending a familial breast clinic (Brain *et al*, 2000). However, although all of the clinics in this study reported that risk counselling was provided as part of the screening programme, some of the women who had participated in the programme for more than 1 year stated they had not been told what their risk levels were. As significantly less well-educated women recalled being told their risk, this may have been due to a lack of understanding of complex information. However, as significantly more high-risk women reported being told their risk level, it could also be a reflection of different counselling services being offered to high-risk compared with moderate-risk women.

Of those who stated that they had been told their risk level, 69% accurately reported the same risk level as the clinic assessment. This is similar to rates reported in other studies (Hopwood *et al*, 2003). Although accurately recalling risk level does not necessarily accord with women's perception of their vulnerability (Hopwood *et al*, 2003), in this study the high-risk women were more likely to believe that they may develop breast cancer in their lifetime.

Another primary motivation for attending a family history clinic is to be advised on strategies to deal with familial risk (Brain *et al*, 2000). For the majority of women, the only strategy available is mammographic surveillance. However, clinicians are concerned about the low sensitivity in younger women with denser breasts. The MARIBS study (Leach *et al*, 2005), which compared the sensitivity of different screening techniques in high-risk women in this age range, reported that mammography has a 40% sensitivity. However, a number of women in this study tended to over-rate the sensitivity of mammography; 20% believed that a normal result meant there was definitely no cancer present and the overwhelming majority did not know that mammographic screening had not been proven to save lives in the younger age group. The absence of definitive proof for mortality reduction from mammography in younger women does by no means constitute proof of absence of such an effect. However, there is a debate in the medical world whether there is proof that screening women aged between 40 and 49 years reduces mortality. Evidence of mortality reduction has come from a number of meta-analyses of trials (Smart *et al*, 1995; Hendrick *et al*, 1997; Kerlikowske, 1997; Moss *et al*, 2006). However, the conclusions have remained controversial for several reasons, including concerns about data that came from trials not specifically designed to evaluate younger women, the quality of the design of some of the trials, the effect of beginning screening at ages just before 50 years and the end points that were measured (Olsen and Gotzsche, 2001). The Age trial (Moss *et al*, 2006), which was designed specifically to measure the effect of screening in younger women, concluded that although there was a reduction in breast cancer mortality in the screened group compared with those not being screened, this was not significant. As they point out, this may be due to the diminished power of the trial (60% to detect a 20% mortality reduction) caused by a smaller than planned sample and a lower than anticipated mortality from breast cancer in the control group, which is probably due to improvements in treatment and consequent survival.

Because women with a family history of breast cancer are at greater risk, in the United Kingdom they are recommended to begin annual mammography screening from the age of 40 years. However, there are theoretical concerns that screening may induce breast cancers in these women, as they may have a genetic susceptibility to radiation, for example, women with BRAC mutations are likely to be more sensitive to ionising radiation as BRAC genes are integral in repairing breast cells (Powell and Kachnic, 2003). They could also comprise a ‘high-dose’ subgroup, as they begin mammography screening at a younger age, have a greater lifetime cumulative exposure to radiation and are more likely to be recalled for further tests. However, no randomised trials have been conducted with this population. Furthermore, as women with a family history of breast cancer are already being offered annual mammography, it would not be feasible to conduct a randomised trial in this population in the United Kingdom. There is. however, a large prospective study ongoing in the United Kingdom (FH01) (Mackay *et al*, 2001), which is due to report in 2010.

In this study, the number of women not attending mammography screening was very small and the main barriers to attendance were practical ones. This reflects the findings in a previous study, which found that once accepted on a family history programme women are highly motivated to attend mammographic screening. Lalloo *et al* (1998) reported that attendance rates at a single UK clinic for first and subsequent screens were 95.2 and 98.9%, respectively. This may be because women with an increased perception of breast cancer risk are more likely to participate in screening in an attempt to gain control over the disease (Katapodi *et al*, 2004). Another possibility could also be that participating in what is generally seen as a health responsible behaviour may be a way of pre-empting feelings of self-blame (Silverman *et al*, 2001) if women do develop breast cancer. This view may be further reinforced by having the option to participate in a screening programme, which is not offered to others in the same age range without an FHBC. The high attendance also suggests that the majority of women with an FHBC may not be making an informed choice when attending mammography screening. Although making an informed choice is recognised as important, there are concerns that given the lack of options available for screening women with an FHBC, knowledge of the uncertainty about its effectiveness in younger women may increase levels of anxiety.

Although this study was large and recruited women from throughout the United Kingdom, there is still a possibility of non-response bias, and the results may not be representative of all women offered the option of participating in a mammographic screening programme because of an FHBC. The strength of the study is that it is the first large multicentre UK study to measure knowledge and views about mammography in women with an FHBC.

In conclusion, once a woman's increased risk level has been confirmed by health professionals, the only strategy for managing this risk for the majority of women is to participate in an annual mammography screening programme despite the uncertainty at present as to the effectiveness of mammography in this group of women. The results from this study highlight that some women have an overly optimistic view of the role mammography may play in reducing breast cancer mortality. Further attention should be given to providing women with an FHBC information about the potential benefits and limitations of mammographic screening, including its lack of sensitivity. It may also be beneficial if the essence of the debate is communicated to women.

## Figures and Tables

**Table 1:** Characteristics of participants summarised by level of risk

	**Level of risk**	
**Characteristics**	**Moderate* N*=1350**	**High* N*=957**
*Sociodemographic*		
Age (median age range)	43 (35–49)^**^	42 (35–49)
Married/living with a partner (%)	1078 (80)	763 (80)
College/university education (%)	413 (31)	283 (30)
White (%)	1290 (96)	920 (96)
Biological children (%)	1090 (81)	763 (80)
Post-menopausal (%)	213 (16)	138 (14)
		
*Family history*		
Relative died from breast cancer (%)	946 (70)^**^	789 (82)
Relative being treated for breast cancer (%)	312 (23)^**^	278 (29)
		
*Screening history*		
First mammogram (%)	198 (15)	121 (13)
First time in screening programme (%)	270 (20)	164 (17)
		
*Cancer worry*		
CWS mean (s.d.)	11.08 (3.06)^**^	11.67 (3.20)
PCQ median (range)	2 (0–36)^**^	3 (0–36)

^**^P<0.01.

**Table 2:** Reasons given for not attending the mammography appointment

**Reasons given**	**Number^a^**	**Main reason^b^**
*Physical constraints*		
1. I had work commitments	5	3
2. I was on holiday.	4	2
3. I had problems attending due to my disability	3	1
4. I was not well	4	2
5. I had to care for a dependent	1	
6. I had problems getting to the breast care unit (includes 13. The times were inconvenient)	8	4
7. I forgot	5	2
20. I have just had a mammogram elsewhere	5	2
		
Other – moved and not referred yet to new clinic	1	
Sub-total	36	
		
*Concern about having a mammogram*		
8. I do not think it is beneficial for me	1	
9. I was worried it would be painful	3	1
10. The benefits of mammography have not been proven	2	
11. The last experience discouraged me	1	
12. Someone else has put me off	1	
14. I was anxious about the possible outcome of screening (i.e., anxious about my results)	7	3
15. I would prefer not to know if something was wrong.	1	1
16. I was worried about the effects of radiation	4	2
17. I don't trust the medical profession	2	1
18. I examine myself for lumps	1	
19. I prefer an alternative approach	1	1
		
Other – Want MRI scans instead of X-rays	1	
Other – Told not high risk	1	1
Other – GP-negative attitude because already had several mammograms	1	1
Sub total	27	
Total	63	

a Women may have given more than one reason for not attending.

b Women may have given up to three main reasons for not attending.
